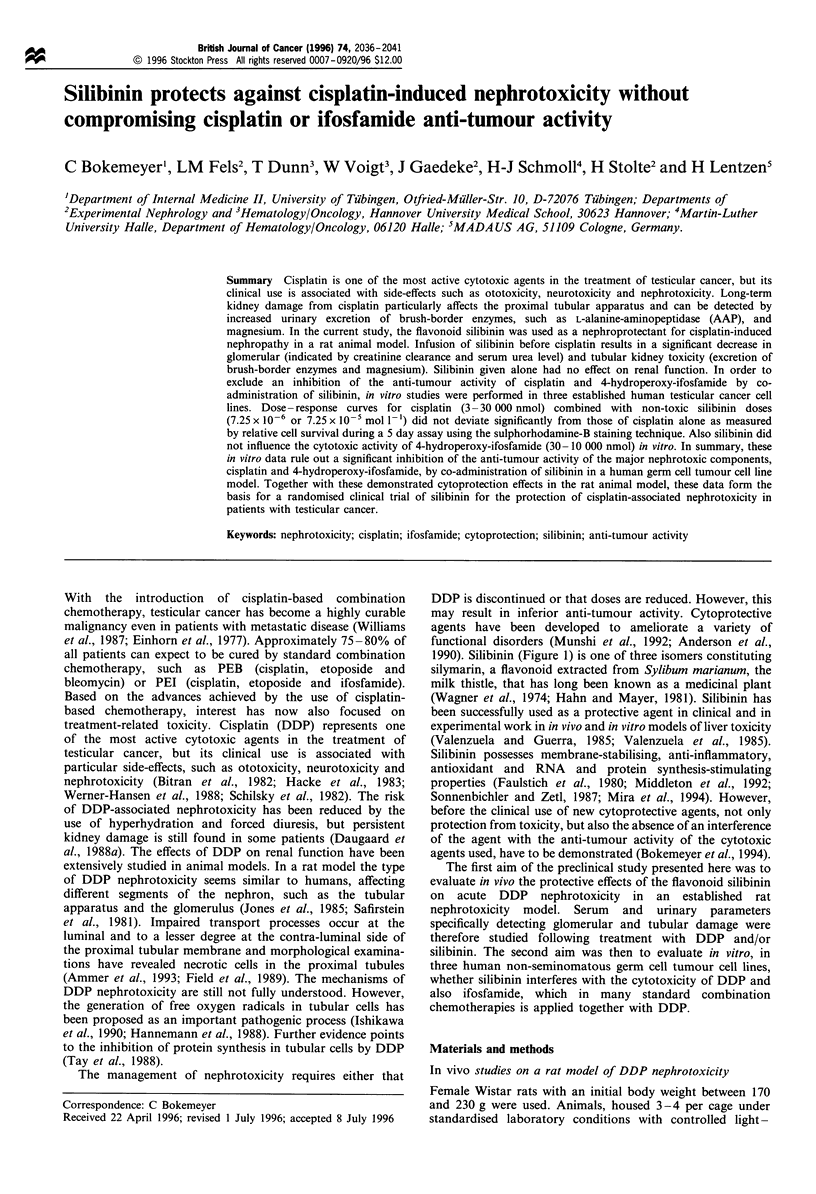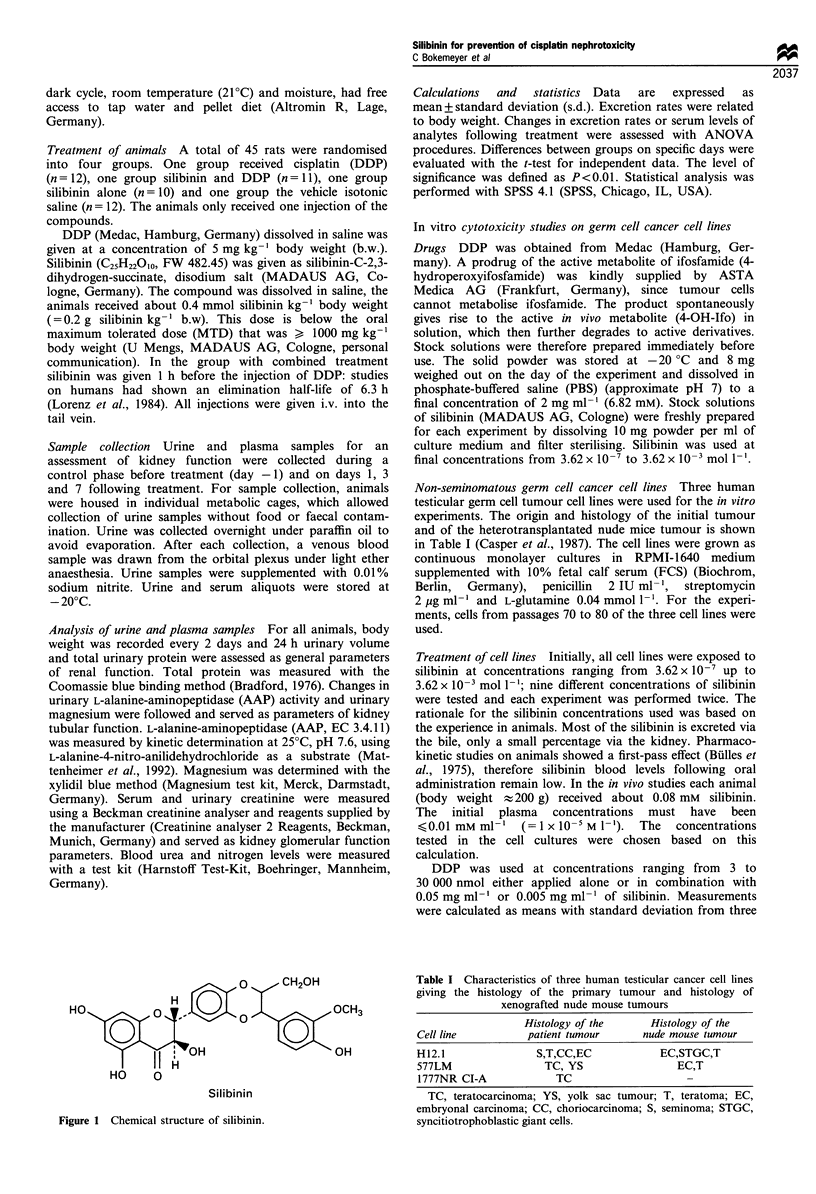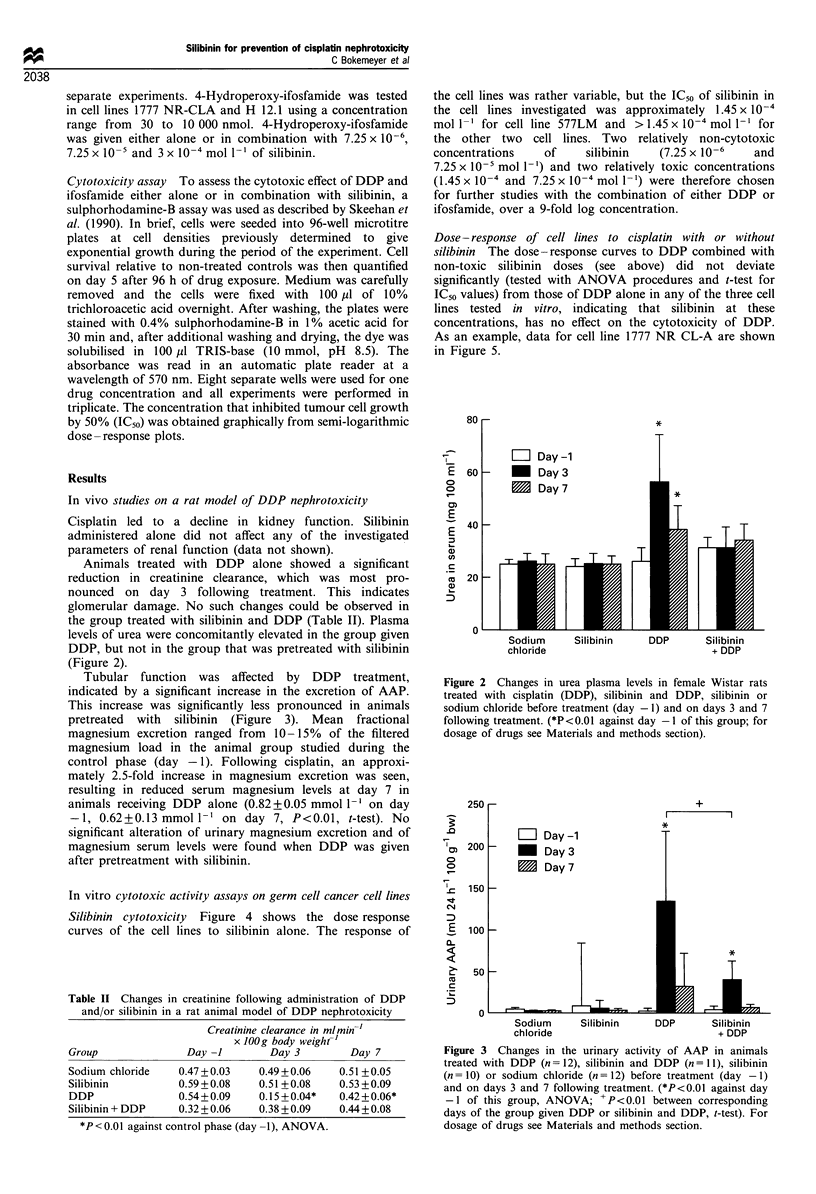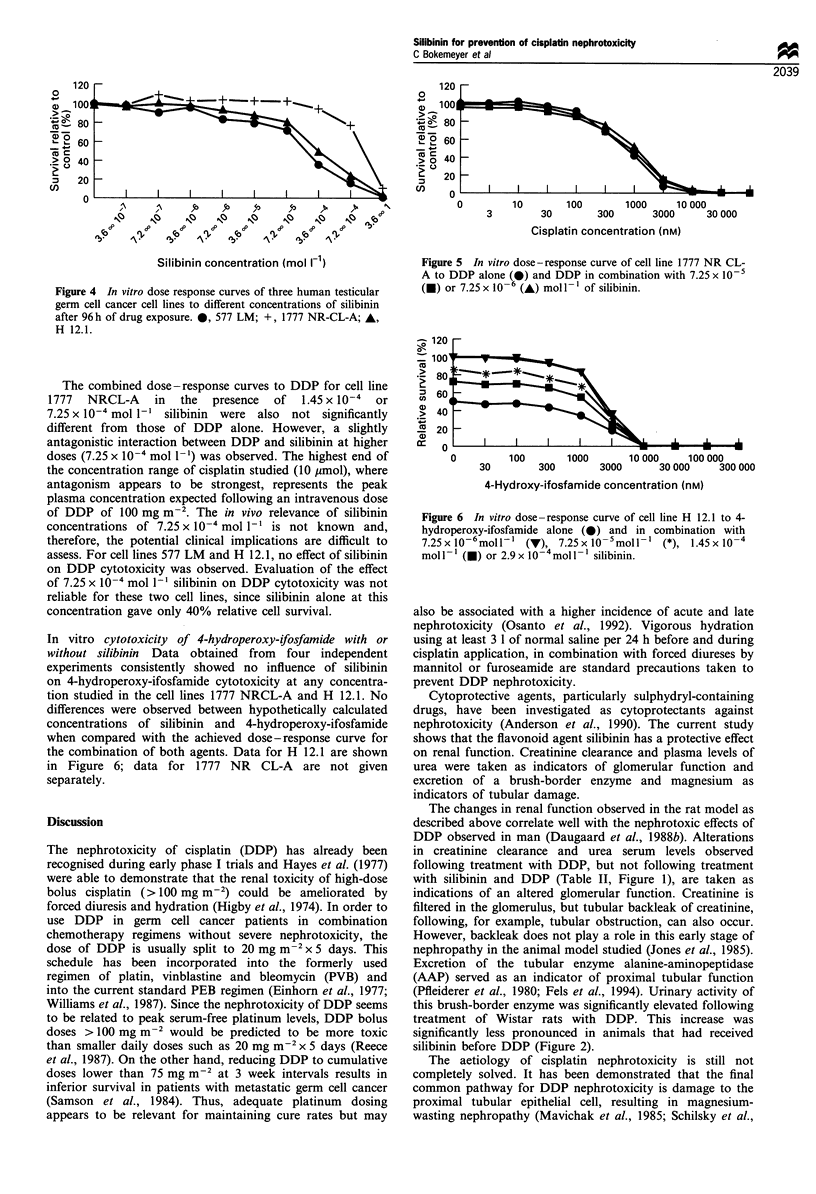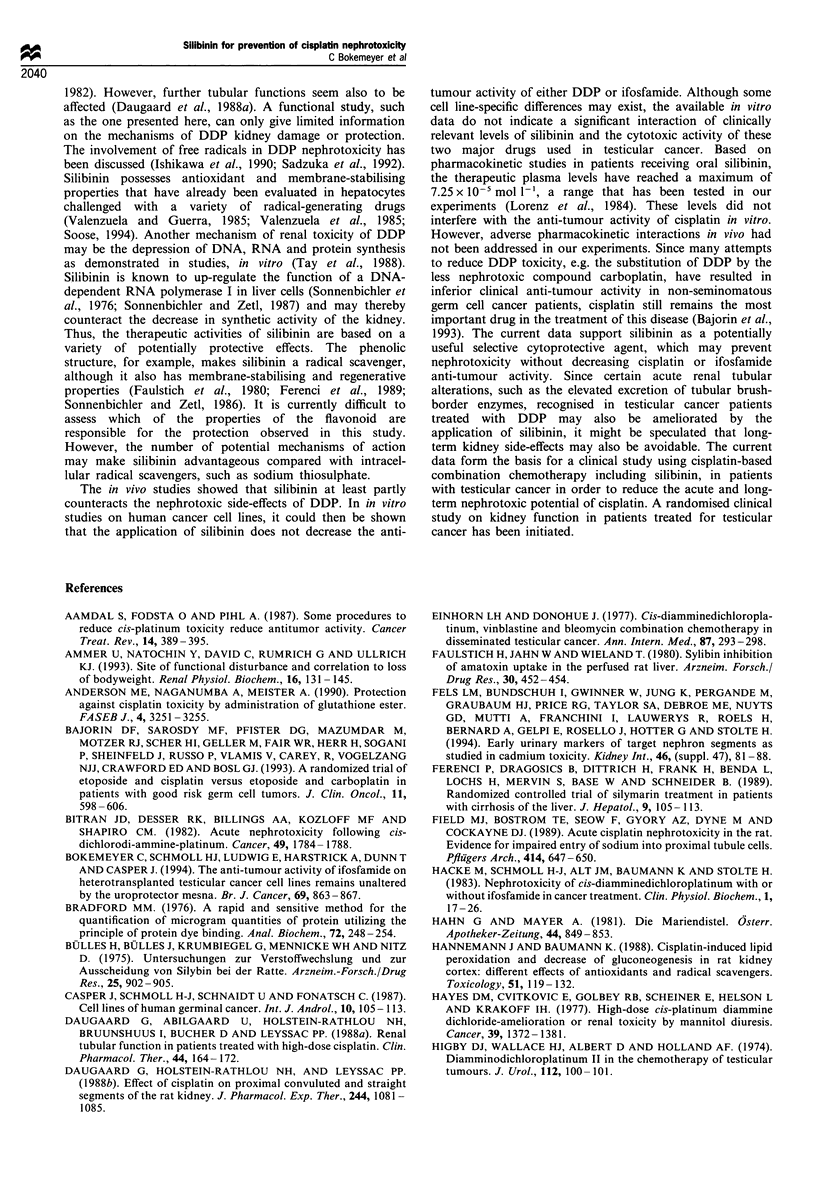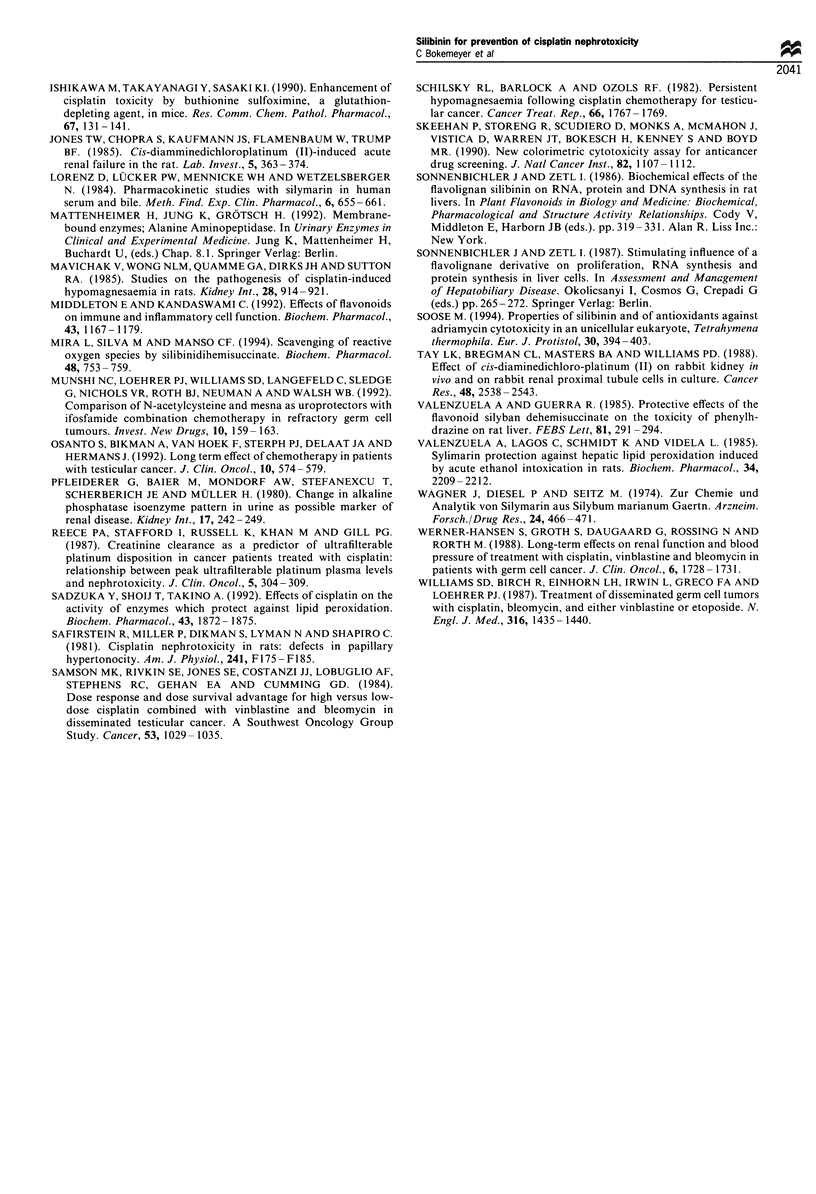# Silibinin protects against cisplatin-induced nephrotoxicity without compromising cisplatin or ifosfamide anti-tumour activity.

**DOI:** 10.1038/bjc.1996.673

**Published:** 1996-12

**Authors:** C. Bokemeyer, L. M. Fels, T. Dunn, W. Voigt, J. Gaedeke, H. J. Schmoll, H. Stolte, H. Lentzen

**Affiliations:** Department of Internal Medicine II, University of Tübingen, Germany.

## Abstract

Cisplatin is one of the most active cytotoxic agents in the treatment of testicular cancer, but its clinical use is associated with side-effects such as ototoxicity, neurotoxicity and nephrotoxicity. Long-term kidney damage from cisplatin particularly affects the proximal tubular apparatus and can be detected by increased urinary excretion of brush-border enzymes, such as L-alanine-aminopeptidase (AAP), and magnesium. In the current study, the flavonoid silibinin was used as a nephroprotectant for cisplatin-induced nephropathy in a rat animal model. Infusion of silibinin before cisplatin results in a significant decrease in glomerular (indicated by creatinine clearance and serum urea level) and tubular kidney toxicity (excretion of brush-border enzymes and magnesium). Silibinin given alone had no effect on renal function. In order to exclude an inhibition of the anti-tumour activity of cisplatin and 4-hydroperoxy-ifosfamide by co-administration of silibinin, in vitro studies were performed in three established human testicular cancer cell lines. Dose-response curves for cisplatin (3-30 000 nmol) combined with non-toxic silibinin doses (7.25 x 10(-6) or 7.25 x 10(-5) mol l-1) did not deviate significantly from those of cisplatin alone as measured by relative cell survival during a 5 day assay using the sulphorhodamine-B staining technique. Also silibinin did not influence the cytotoxic activity of 4-hydroperoxy-ifosfamide (30-10 000 nmol) in vitro. In summary, these in vitro data rule out a significant inhibition of the anti-tumour activity of the major nephrotoxic components, cisplatin and 4-hydroperoxy-ifosfamide, by co-administration of silibinin in a human germ cell tumour cell line model. Together with these demonstrated cytoprotection effects in the rat animal model, these data form the basis for a randomised clinical trial of silibinin for the protection of cisplatin-associated nephrotoxicity in patients with testicular cancer.


British Journal of Cancer (1996) 74, 2036-2041
© 1996 Stockton Press All rights reserved 0007-0920/96 $12.00

### Silibinin protects against cisplatin-induced nephrotoxicity without compromising cisplatin or ifosfamide anti-tumour activity

C Bokemeyer[1], LM Fels[2], T Dunn[3], W Voigt[3], J Gaedeke[2], H-J Schmoll[4], H Stolte[2] and H Lentzen[5]

[1]*Department of Internal Medicine II, University of Tübingen, Otfried-Müller-Str. 10, D-72076 Tübingen; Departments of* [2]*Experimental Nephrology and* [3]*Hematology/Oncology, Hannover University Medical School, 30623 Hannover;* [4]*Martin-Luther University Halle, Department of Hematology/Oncology, 06120 Halle;* [5]*MADAUS AG, 51109 Cologne, Germany.*

**Summary** Cisplatin is one of the most active cytotoxic agents in the treatment of testicular cancer, but its clinical use is associated with side-effects such as ototoxicity, neurotoxicity and nephrotoxicity. Long-term kidney damage from cisplatin particularly affects the proximal tubular apparatus and can be detected by increased urinary excretion of brush-border enzymes, such as L-alanine-aminopeptidase (AAP), and magnesium. In the current study, the flavonoid silibinin was used as a nephroprotectant for cisplatin-induced nephropathy in a rat animal model. Infusion of silibinin before cisplatin results in a significant decrease in glomerular (indicated by creatinine clearance and serum urea level) and tubular kidney toxicity (excretion of brush-border enzymes and magnesium). Silibinin given alone had no effect on renal function. In order to exclude an inhibition of the anti-tumour activity of cisplatin and 4-hydroperoxy-ifosfamide by co-administration of silibinin, *in vitro* studies were performed in three established human testicular cancer cell lines. Dose–response curves for cisplatin (3–30 000 nmol) combined with non-toxic silibinin doses ($7.25 \times 10^{-6}$ or $7.25 \times 10^{-5}$ mol $l^{-1}$) did not deviate significantly from those of cisplatin alone as measured by relative cell survival during a 5 day assay using the sulphorhodamine-B staining technique. Also silibinin did not influence the cytotoxic activity of 4-hydroperoxy-ifosfamide (30–10 000 nmol) *in vitro*. In summary, these *in vitro* data rule out a significant inhibition of the anti-tumour activity of the major nephrotoxic components, cisplatin and 4-hydroperoxy-ifosfamide, by co-administration of silibinin in a human germ cell tumour cell line model. Together with these demonstrated cytoprotection effects in the rat animal model, these data form the basis for a randomised clinical trial of silibinin for the protection of cisplatin-associated nephrotoxicity in patients with testicular cancer.

**Keywords:** nephrotoxicity; cisplatin; ifosfamide; cytoprotection; silibinin; anti-tumour activity

With the introduction of cisplatin-based combination chemotherapy, testicular cancer has become a highly curable malignancy even in patients with metastatic disease (Williams *et al.*, 1987; Einhorn *et al.*, 1977). Approximately 75–80% of all patients can expect to be cured by standard combination chemotherapy, such as PEB (cisplatin, etoposide and bleomycin) or PEI (cisplatin, etoposide and ifosfamide). Based on the advances achieved by the use of cisplatin-based chemotherapy, interest has now also focused on treatment-related toxicity. Cisplatin (DDP) represents one of the most active cytotoxic agents in the treatment of testicular cancer, but its clinical use is associated with particular side-effects, such as ototoxicity, neurotoxicity and nephrotoxicity (Bitran *et al.*, 1982; Hacke *et al.*, 1983; Werner-Hansen *et al.*, 1988; Schilsky *et al.*, 1982). The risk of DDP-associated nephrotoxicity has been reduced by the use of hyperhydration and forced diuresis, but persistent kidney damage is still found in some patients (Daugaard *et al.*, 1988*a*). The effects of DDP on renal function have been extensively studied in animal models. In a rat model the type of DDP nephrotoxicity seems similar to humans, affecting different segments of the nephron, such as the tubular apparatus and the glomerulus (Jones *et al.*, 1985; Safirstein *et al.*, 1981). Impaired transport processes occur at the luminal and to a lesser degree at the contra-luminal side of the proximal tubular membrane and morphological examinations have revealed necrotic cells in the proximal tubules (Ammer *et al.*, 1993; Field *et al.*, 1989). The mechanisms of DDP nephrotoxicity are still not fully understood. However, the generation of free oxygen radicals in tubular cells has been proposed as an important pathogenic process (Ishikawa *et al.*, 1990; Hannemann *et al.*, 1988). Further evidence points to the inhibition of protein synthesis in tubular cells by DDP (Tay *et al.*, 1988).

The management of nephrotoxicity requires either that DDP is discontinued or that doses are reduced. However, this may result in inferior anti-tumour activity. Cytoprotective agents have been developed to ameliorate a variety of functional disorders (Munshi *et al.*, 1992; Anderson *et al.*, 1990). Silibinin (Figure 1) is one of three isomers constituting silymarin, a flavonoid extracted from *Sylibum marianum*, the milk thistle, that has long been known as a medicinal plant (Wagner *et al.*, 1974; Hahn and Mayer, 1981). Silibinin has been successfully used as a protective agent in clinical and in experimental work in *in vivo* and *in vitro* models of liver toxicity (Valenzuela and Guerra, 1985; Valenzuela *et al.*, 1985). Silibinin possesses membrane-stabilising, anti-inflammatory, antioxidant and RNA and protein synthesis-stimulating properties (Faulstich *et al.*, 1980; Middleton *et al.*, 1992; Sonnenbichler and Zetl, 1987; Mira *et al.*, 1994). However, before the clinical use of new cytoprotective agents, not only protection from toxicity, but also the absence of an interference of the agent with the anti-tumour activity of the cytotoxic agents used, have to be demonstrated (Bokemeyer *et al.*, 1994).

The first aim of the preclinical study presented here was to evaluate *in vivo* the protective effects of the flavonoid silibinin on acute DDP nephrotoxicity in an established rat nephrotoxicity model. Serum and urinary parameters specifically detecting glomerular and tubular damage were therefore studied following treatment with DDP and/or silibinin. The second aim was then to evaluate *in vitro*, in three human non-seminomatous germ cell tumour cell lines, whether silibinin interferes with the cytotoxicity of DDP and also ifosfamide, which in many standard combination chemotherapies is applied together with DDP.

### Materials and methods

### In vivo *studies on a rat model of DDP nephrotoxicity*

Female Wistar rats with an initial body weight between 170 and 230 g were used. Animals, housed 3–4 per cage under standardised laboratory conditions with controlled light–

Correspondence: C Bokemeyer
Received 22 April 1996; revised 1 July 1996; accepted 8 July 1996

dark cycle, room temperature (21°C) and moisture, had free access to tap water and pellet diet (Altromin R, Lage, Germany).

*Treatment of animals* A total of 45 rats were randomised into four groups. One group received cisplatin (DDP) ($n=12$), one group silibinin and DDP ($n=11$), one group silibinin alone ($n=10$) and one group the vehicle isotonic saline ($n=12$). The animals only received one injection of the compounds.

DDP (Medac, Hamburg, Germany) dissolved in saline was given at a concentration of 5 mg kg$^{-1}$ body weight (b.w.). Silibinin ($C_{25}H_{22}O_{10}$, FW 482.45) was given as silibinin-C-2,3-dihydrogen-succinate, disodium salt (MADAUS AG, Cologne, Germany). The compound was dissolved in saline, the animals received about 0.4 mmol silibinin kg$^{-1}$ body weight (=0.2 g silibinin kg$^{-1}$ b.w). This dose is below the oral maximum tolerated dose (MTD) that was $\geqslant$ 1000 mg kg$^{-1}$ body weight (U Mengs, MADAUS AG, Cologne, personal communication). In the group with combined treatment silibinin was given 1 h before the injection of DDP: studies on humans had shown an elimination half-life of 6.3 h (Lorenz *et al.*, 1984). All injections were given i.v. into the tail vein.

*Sample collection* Urine and plasma samples for an assessment of kidney function were collected during a control phase before treatment (day −1) and on days 1, 3 and 7 following treatment. For sample collection, animals were housed in individual metabolic cages, which allowed collection of urine samples without food or faecal contamination. Urine was collected overnight under paraffin oil to avoid evaporation. After each collection, a venous blood sample was drawn from the orbital plexus under light ether anaesthesia. Urine samples were supplemented with 0.01% sodium nitrite. Urine and serum aliquots were stored at −20°C.

*Analysis of urine and plasma samples* For all animals, body weight was recorded every 2 days and 24 h urinary volume and total urinary protein were assessed as general parameters of renal function. Total protein was measured with the Coomassie blue binding method (Bradford, 1976). Changes in urinary L-alanine-aminopeptidase (AAP) activity and urinary magnesium were followed and served as parameters of kidney tubular function. L-alanine-aminopeptidase (AAP, EC 3.4.11) was measured by kinetic determination at 25°C, pH 7.6, using L-alanine-4-nitro-anilidehydrochloride as a substrate (Mattenheimer *et al.*, 1992). Magnesium was determined with the xylidil blue method (Magnesium test kit, Merck, Darmstadt, Germany). Serum and urinary creatinine were measured using a Beckman creatinine analyser and reagents supplied by the manufacturer (Creatinine analyser 2 Reagents, Beckman, Munich, Germany) and served as kidney glomerular function parameters. Blood urea and nitrogen levels were measured with a test kit (Harnstoff Test-Kit, Boehringer, Mannheim, Germany).

*Calculations and statistics* Data are expressed as mean ± standard deviation (s.d.). Excretion rates were related to body weight. Changes in excretion rates or serum levels of analytes following treatment were assessed with ANOVA procedures. Differences between groups on specific days were evaluated with the *t*-test for independent data. The level of significance was defined as $P<0.01$. Statistical analysis was performed with SPSS 4.1 (SPSS, Chicago, IL, USA).

### In vitro *cytotoxicity studies on germ cell cancer cell lines*

*Drugs* DDP was obtained from Medac (Hamburg, Germany). A prodrug of the active metabolite of ifosfamide (4-hydroperoxyifosfamide) was kindly supplied by ASTA Medica AG (Frankfurt, Germany), since tumour cells cannot metabolise ifosfamide. The product spontaneously gives rise to the active *in vivo* metabolite (4-OH-Ifo) in solution, which then further degrades to active derivatives. Stock solutions were therefore prepared immediately before use. The solid powder was stored at −20 °C and 8 mg weighed out on the day of the experiment and dissolved in phosphate-buffered saline (PBS) (approximate pH 7) to a final concentration of 2 mg ml$^{-1}$ (6.82 mM). Stock solutions of silibinin (MADAUS AG, Cologne) were freshly prepared for each experiment by dissolving 10 mg powder per ml of culture medium and filter sterilising. Silibinin was used at final concentrations from $3.62\times10^{-7}$ to $3.62\times10^{-3}$ mol l$^{-1}$.

*Non-seminomatous germ cell cancer cell lines* Three human testicular germ cell tumour cell lines were used for the *in vitro* experiments. The origin and histology of the initial tumour and of the heterotransplantated nude mice tumour is shown in Table I (Casper *et al.*, 1987). The cell lines were grown as continuous monolayer cultures in RPMI-1640 medium supplemented with 10% fetal calf serum (FCS) (Biochrom, Berlin, Germany), penicillin 2 IU ml$^{-1}$, streptomycin 2 $\mu$g ml$^{-1}$ and L-glutamine 0.04 mmol l$^{-1}$. For the experiments, cells from passages 70 to 80 of the three cell lines were used.

*Treatment of cell lines* Initially, all cell lines were exposed to silibinin at concentrations ranging from $3.62\times10^{-7}$ up to $3.62\times10^{-3}$ mol l$^{-1}$; nine different concentrations of silibinin were tested and each experiment was performed twice. The rationale for the silibinin concentrations used was based on the experience in animals. Most of the silibinin is excreted via the bile, only a small percentage via the kidney. Pharmacokinetic studies on animals showed a first-pass effect (Bülles *et al.*, 1975), therefore silibinin blood levels following oral administration remain low. In the *in vivo* studies each animal (body weight ≈200 g) received about 0.08 mM silibinin. The initial plasma concentrations must have been $\leqslant$0.01 mM ml$^{-1}$ (=$1\times10^{-5}$ M l$^{-1}$). The concentrations tested in the cell cultures were chosen based on this calculation.

DDP was used at concentrations ranging from 3 to 30 000 nmol either applied alone or in combination with 0.05 mg ml$^{-1}$ or 0.005 mg ml$^{-1}$ of silibinin. Measurements were calculated as means with standard deviation from three

**Figure 1** Chemical structure of silibinin.

**Table I** Characteristics of three human testicular cancer cell lines giving the histology of the primary tumour and histology of xenografted nude mouse tumours

| Cell line | *Histology of the patient tumour* | *Histology of the nude mouse tumour* |
|---|---|---|
| H12.1 | S,T,CC,EC | EC,STGC,T |
| 577LM | TC, YS | EC,T |
| 1777NR CI-A | TC | – |

TC, teratocarcinoma; YS, yolk sac tumour; T, teratoma; EC, embryonal carcinoma; CC, choriocarcinoma; S, seminoma; STGC, syncitiotrophoblastic giant cells.

separate experiments. 4-Hydroperoxy-ifosfamide was tested in cell lines 1777 NR-CLA and H 12.1 using a concentration range from 30 to 10 000 nmol. 4-Hydroperoxy-ifosfamide was given either alone or in combination with $7.25 \times 10^{-6}$, $7.25 \times 10^{-5}$ and $3 \times 10^{-4}$ mol $l^{-1}$ of silibinin.

*Cytotoxicity assay* To assess the cytotoxic effect of DDP and ifosfamide either alone or in combination with silibinin, a sulphorhodamine-B assay was used as described by Skeehan *et al.* (1990). In brief, cells were seeded into 96-well microtitre plates at cell densities previously determined to give exponential growth during the period of the experiment. Cell survival relative to non-treated controls was then quantified on day 5 after 96 h of drug exposure. Medium was carefully removed and the cells were fixed with 100 $\mu$l of 10% trichloroacetic acid overnight. After washing, the plates were stained with 0.4% sulphorhodamine-B in 1% acetic acid for 30 min and, after additional washing and drying, the dye was solubilised in 100 $\mu$l TRIS-base (10 mmol, pH 8.5). The absorbance was read in an automatic plate reader at a wavelength of 570 nm. Eight separate wells were used for one drug concentration and all experiments were performed in triplicate. The concentration that inhibited tumour cell growth by 50% ($IC_{50}$) was obtained graphically from semi-logarithmic dose–response plots.

### Results

### In vivo *studies on a rat model of DDP nephrotoxicity*

Cisplatin led to a decline in kidney function. Silibinin administered alone did not affect any of the investigated parameters of renal function (data not shown).

Animals treated with DDP alone showed a significant reduction in creatinine clearance, which was most pronounced on day 3 following treatment. This indicates glomerular damage. No such changes could be observed in the group treated with silibinin and DDP (Table II). Plasma levels of urea were concomitantly elevated in the group given DDP, but not in the group that was pretreated with silibinin (Figure 2).

Tubular function was affected by DDP treatment, indicated by a significant increase in the excretion of AAP. This increase was significantly less pronounced in animals pretreated with silibinin (Figure 3). Mean fractional magnesium excretion ranged from 10–15% of the filtered magnesium load in the animal group studied during the control phase (day −1). Following cisplatin, an approximately 2.5-fold increase in magnesium excretion was seen, resulting in reduced serum magnesium levels at day 7 in animals receiving DDP alone ($0.82 \pm 0.05$ mmol $l^{-1}$ on day −1, $0.62 \pm 0.13$ mmol $l^{-1}$ on day 7, $P < 0.01$, $t$-test). No significant alteration of urinary magnesium excretion and of magnesium serum levels were found when DDP was given after pretreatment with silibinin.

### In vitro *cytotoxic activity assays on germ cell cancer cell lines*

*Silibinin cytotoxicity* Figure 4 shows the dose response curves of the cell lines to silibinin alone. The response of the cell lines was rather variable, but the $IC_{50}$ of silibinin in the cell lines investigated was approximately $1.45 \times 10^{-4}$ mol $l^{-1}$ for cell line 577LM and $> 1.45 \times 10^{-4}$ mol $l^{-1}$ for the other two cell lines. Two relatively non-cytotoxic concentrations of silibinin ($7.25 \times 10^{-6}$ and $7.25 \times 10^{-5}$ mol $l^{-1}$) and two relatively toxic concentrations ($1.45 \times 10^{-4}$ and $7.25 \times 10^{-4}$ mol $l^{-1}$) were therefore chosen for further studies with the combination of either DDP or ifosfamide, over a 9-fold log concentration.

*Dose–response of cell lines to cisplatin with or without silibinin* The dose–response curves to DDP combined with non-toxic silibinin doses (see above) did not deviate significantly (tested with ANOVA procedures and $t$-test for $IC_{50}$ values) from those of DDP alone in any of the three cell lines tested *in vitro*, indicating that silibinin at these concentrations, has no effect on the cytotoxicity of DDP. As an example, data for cell line 1777 NR CL-A are shown in Figure 5.

**Table II** Changes in creatinine following administration of DDP and/or silibinin in a rat animal model of DDP nephrotoxicity

| *Group* | *Creatinine clearance in ml min⁻¹ × 100 g body weight⁻¹* Day −1 | Day 3 | Day 7 |
|---|---|---|---|
| Sodium chloride | $0.47 \pm 0.03$ | $0.49 \pm 0.06$ | $0.51 \pm 0.05$ |
| Silibinin | $0.59 \pm 0.08$ | $0.51 \pm 0.08$ | $0.53 \pm 0.09$ |
| DDP | $0.54 \pm 0.09$ | $0.15 \pm 0.04$* | $0.42 \pm 0.06$* |
| Silibinin + DDP | $0.32 \pm 0.06$ | $0.38 \pm 0.09$ | $0.44 \pm 0.08$ |

*$P < 0.01$ against control phase (day −1), ANOVA.

**Figure 2** Changes in urea plasma levels in female Wistar rats treated with cisplatin (DDP), silibinin and DDP, silibinin or sodium chloride before treatment (day −1) and on days 3 and 7 following treatment. (*$P < 0.01$ against day −1 of this group; for dosage of drugs see Materials and methods section).

**Figure 3** Changes in the urinary activity of AAP in animals treated with DDP ($n = 12$), silibinin and DDP ($n = 11$), silibinin ($n = 10$) or sodium chloride ($n = 12$) before treatment (day −1) and on days 3 and 7 following treatment. (*$P < 0.01$ against day −1 of this group, ANOVA; $^{+}P < 0.01$ between corresponding days of the group given DDP or silibinin and DDP, $t$-test). For dosage of drugs see Materials and methods section.

**Figure 4** *In vitro* dose response curves of three human testicular germ cell cancer cell lines to different concentrations of silibinin after 96 h of drug exposure. ●, 577 LM; +, 1777 NR-CL-A; ▲, H 12.1.

The combined dose–response curves to DDP for cell line 1777 NRCL-A in the presence of $1.45\times10^{-4}$ or $7.25\times10^{-4}$ mol $l^{-1}$ silibinin were also not significantly different from those of DDP alone. However, a slightly antagonistic interaction between DDP and silibinin at higher doses ($7.25\times10^{-4}$ mol $l^{-1}$) was observed. The highest end of the concentration range of cisplatin studied (10 $\mu$mol), where antagonism appears to be strongest, represents the peak plasma concentration expected following an intravenous dose of DDP of 100 mg $m^{-2}$. The *in vivo* relevance of silibinin concentrations of $7.25\times10^{-4}$ mol $l^{-1}$ is not known and, therefore, the potential clinical implications are difficult to assess. For cell lines 577 LM and H 12.1, no effect of silibinin on DDP cytotoxicity was observed. Evaluation of the effect of $7.25\times10^{-4}$ mol $l^{-1}$ silibinin on DDP cytotoxicity was not reliable for these two cell lines, since silibinin alone at this concentration gave only 40% relative cell survival.

In vitro *cytotoxicity of 4-hydroperoxy-ifosfamide with or without silibinin* Data obtained from four independent experiments consistently showed no influence of silibinin on 4-hydroperoxy-ifosfamide cytotoxicity at any concentration studied in the cell lines 1777 NRCL-A and H 12.1. No differences were observed between hypothetically calculated concentrations of silibinin and 4-hydroperoxy-ifosfamide when compared with the achieved dose–response curve for the combination of both agents. Data for H 12.1 are shown in Figure 6; data for 1777 NR CL-A are not given separately.

### Discussion

The nephrotoxicity of cisplatin (DDP) has already been recognised during early phase I trials and Hayes *et al.* (1977) were able to demonstrate that the renal toxicity of high-dose bolus cisplatin (>100 mg $m^{-2}$) could be ameliorated by forced diuresis and hydration (Higby *et al.*, 1974). In order to use DDP in germ cell cancer patients in combination chemotherapy regimens without severe nephrotoxicity, the dose of DDP is usually split to 20 mg $m^{-2}\times5$ days. This schedule has been incorporated into the formerly used regimen of platin, vinblastine and bleomycin (PVB) and into the current standard PEB regimen (Einhorn *et al.*, 1977; Williams *et al.*, 1987). Since the nephrotoxicity of DDP seems to be related to peak serum-free platinum levels, DDP bolus doses >100 mg $m^{-2}$ would be predicted to be more toxic than smaller daily doses such as 20 mg $m^{-2}\times5$ days (Reece *et al.*, 1987). On the other hand, reducing DDP to cumulative doses lower than 75 mg $m^{-2}$ at 3 week intervals results in inferior survival in patients with metastatic germ cell cancer (Samson *et al.*, 1984). Thus, adequate platinum dosing appears to be relevant for maintaining cure rates but may

**Figure 5** *In vitro* dose–response curve of cell line 1777 NR CL-A to DDP alone (●) and DDP in combination with $7.25\times10^{-5}$ (■) or $7.25\times10^{-6}$ (▲) mol $l^{-1}$ of silibinin.

**Figure 6** *In vitro* dose–response curve of cell line H 12.1 to 4-hydroperoxy-ifosfamide alone (●) and in combination with $7.25\times10^{-6}$ mol $l^{-1}$ (▼), $7.25\times10^{-5}$ mol $l^{-1}$ (*), $1.45\times10^{-4}$ mol $l^{-1}$ (■) or $2.9\times10^{-4}$ mol $l^{-1}$ silibinin.

also be associated with a higher incidence of acute and late nephrotoxicity (Osanto *et al.*, 1992). Vigorous hydration using at least 3 l of normal saline per 24 h before and during cisplatin application, in combination with forced diureses by mannitol or furoseamide are standard precautions taken to prevent DDP nephrotoxicity.

Cytoprotective agents, particularly sulphydryl-containing drugs, have been investigated as cytoprotectants against nephrotoxicity (Anderson *et al.*, 1990). The current study shows that the flavonoid agent silibinin has a protective effect on renal function. Creatinine clearance and plasma levels of urea were taken as indicators of glomerular function and excretion of a brush-border enzyme and magnesium as indicators of tubular damage.

The changes in renal function observed in the rat model as described above correlate well with the nephrotoxic effects of DDP observed in man (Daugaard *et al.*, 1988*b*). Alterations in creatinine clearance and urea serum levels observed following treatment with DDP, but not following treatment with silibinin and DDP (Table II, Figure 1), are taken as indications of an altered glomerular function. Creatinine is filtered in the glomerulus, but tubular backleak of creatinine, following, for example, tubular obstruction, can also occur. However, backleak does not play a role in this early stage of nephropathy in the animal model studied (Jones *et al.*, 1985). Excretion of the tubular enzyme alanine-aminopeptidase (AAP) served as an indicator of proximal tubular function (Pfleiderer *et al.*, 1980; Fels *et al.*, 1994). Urinary activity of this brush-border enzyme was significantly elevated following treatment of Wistar rats with DDP. This increase was significantly less pronounced in animals that had received silibinin before DDP (Figure 2).

The aetiology of cisplatin nephrotoxicity is still not completely solved. It has been demonstrated that the final common pathway for DDP nephrotoxicity is damage to the proximal tubular epithelial cell, resulting in magnesium-wasting nephropathy (Mavichak *et al.*, 1985; Schilsky *et al.*,

1982). However, further tubular functions seem also to be affected (Daugaard *et al*., 1988*a*). A functional study, such as the one presented here, can only give limited information on the mechanisms of DDP kidney damage or protection. The involvement of free radicals in DDP nephrotoxicity has been discussed (Ishikawa *et al*., 1990; Sadzuka *et al*., 1992). Silibinin possesses antioxidant and membrane-stabilising properties that have already been evaluated in hepatocytes challenged with a variety of radical-generating drugs (Valenzuela and Guerra, 1985; Valenzuela *et al*., 1985; Soose, 1994). Another mechanism of renal toxicity of DDP may be the depression of DNA, RNA and protein synthesis as demonstrated in studies, *in vitro* (Tay *et al*., 1988). Silibinin is known to up-regulate the function of a DNA-dependent RNA polymerase I in liver cells (Sonnenbichler *et al*., 1976; Sonnenbichler and Zetl, 1987) and may thereby counteract the decrease in synthetic activity of the kidney. Thus, the therapeutic activities of silibinin are based on a variety of potentially protective effects. The phenolic structure, for example, makes silibinin a radical scavenger, although it also has membrane-stabilising and regenerative properties (Faulstich *et al*., 1980; Ferenci *et al*., 1989; Sonnenbichler and Zetl, 1986). It is currently difficult to assess which of the properties of the flavonoid are responsible for the protection observed in this study. However, the number of potential mechanisms of action may make silibinin advantageous compared with intracellular radical scavengers, such as sodium thiosulphate.

The *in vivo* studies showed that silibinin at least partly counteracts the nephrotoxic side-effects of DDP. In *in vitro* studies on human cancer cell lines, it could then be shown that the application of silibinin does not decrease the anti-tumour activity of either DDP or ifosfamide. Although some cell line-specific differences may exist, the available *in vitro* data do not indicate a significant interaction of clinically relevant levels of silibinin and the cytotoxic activity of these two major drugs used in testicular cancer. Based on pharmacokinetic studies in patients receiving oral silibinin, the therapeutic plasma levels have reached a maximum of $7.25 \times 10^{-5}$ mol $l^{-1}$, a range that has been tested in our experiments (Lorenz *et al*., 1984). These levels did not interfere with the anti-tumour activity of cisplatin *in vitro*. However, adverse pharmacokinetic interactions *in vivo* had not been addressed in our experiments. Since many attempts to reduce DDP toxicity, e.g. the substitution of DDP by the less nephrotoxic compound carboplatin, have resulted in inferior clinical anti-tumour activity in non-seminomatous germ cell cancer patients, cisplatin still remains the most important drug in the treatment of this disease (Bajorin *et al*., 1993). The current data support silibinin as a potentially useful selective cytoprotective agent, which may prevent nephrotoxicity without decreasing cisplatin or ifosfamide anti-tumour activity. Since certain acute renal tubular alterations, such as the elevated excretion of tubular brush-border enzymes, recognised in testicular cancer patients treated with DDP may also be ameliorated by the application of silibinin, it might be speculated that long-term kidney side-effects may also be avoidable. The current data form the basis for a clinical study using cisplatin-based combination chemotherapy including silibinin, in patients with testicular cancer in order to reduce the acute and long-term nephrotoxic potential of cisplatin. A randomised clinical study on kidney function in patients treated for testicular cancer has been initiated.

### References

AAMDAL S, FODSTA O AND PIHL A. (1987). Some procedures to reduce *cis*-platinum toxicity reduce antitumor activity. *Cancer Treat. Rev*., **14**, 389–395.

AMMER U, NATOCHIN Y, DAVID C, RUMRICH G AND ULLRICH KJ. (1993). Site of functional disturbance and correlation to loss of bodyweight. *Renal Physiol. Biochem*., **16**, 131–145.

ANDERSON ME, NAGANUMBA A, MEISTER A. (1990). Protection against cisplatin toxicity by administration of glutathione ester. *FASEB J*., **4**, 3251–3255.

BAJORIN DF, SAROSDY MF, PFISTER DG, MAZUMDAR M, MOTZER RJ, SCHER HI, GELLER M, FAIR WR, HERR H, SOGANI P, SHEINFELD J, RUSSO P, VLAMIS V, CAREY, R, VOGELZANG NJJ, CRAWFORD ED AND BOSL GJ. (1993). A randomized trial of etoposide and cisplatin versus etoposide and carboplatin in patients with good risk germ cell tumors. *J. Clin. Oncol*., **11**, 598–606.

BITRAN JD, DESSER RK, BILLINGS AA, KOZLOFF MF AND SHAPIRO CM. (1982). Acute nephrotoxicity following *cis*-dichlorodi-ammine-platinum. *Cancer*, **49**, 1784–1788.

BOKEMEYER C, SCHMOLL HJ, LUDWIG E, HARSTRICK A, DUNN T AND CASPER J. (1994). The anti-tumour activity of ifosfamide on heterotransplanted testicular cancer cell lines remains unaltered by the uroprotector mesna. *Br. J. Cancer*, **69**, 863–867.

BRADFORD MM. (1976). A rapid and sensitive method for the quantification of microgram quantities of protein utilizing the principle of protein dye binding. *Anal. Biochem*., **72**, 248–254.

BÜLLES H, BÜLLES J, KRUMBIEGEL G, MENNICKE WH AND NITZ D. (1975). Untersuchungen zur Verstoffwechslung und zur Ausscheidung von Silybin bei der Ratte. *Arzneim.-Forsch./Drug Res*., **25**, 902–905.

CASPER J, SCHMOLL H-J, SCHNAIDT U AND FONATSCH C. (1987). Cell lines of human germinal cancer. *Int. J. Androl*., **10**, 105–113.

DAUGAARD G, ABILGAARD U, HOLSTEIN-RATHLOU NH, BRUUNSHUUS I, BUCHER D AND LEYSSAC PP. (1988*a*). Renal tubular function in patients treated with high-dose cisplatin. *Clin. Pharmacol. Ther*., **44**, 164–172.

DAUGAARD G, HOLSTEIN-RATHLOU NH, AND LEYSSAC PP. (1988*b*). Effect of cisplatin on proximal convuluted and straight segments of the rat kidney. *J. Pharmacol. Exp. Ther*., **244**, 1081–1085.

EINHORN LH AND DONOHUE J. (1977). *Cis*-diamminedichloroplatinum, vinblastine and bleomycin combination chemotherapy in disseminated testicular cancer. *Ann. Intern. Med*., **87**, 293–298.

FAULSTICH H, JAHN W AND WIELAND T. (1980). Sylibin inhibition of amatoxin uptake in the perfused rat liver. *Arzneim. Forsch./Drug Res*., **30**, 452–454.

FELS LM, BUNDSCHUH I, GWINNER W, JUNG K, PERGANDE M, GRAUBAUM HJ, PRICE RG, TAYLOR SA, DEBROE ME, NUYTS GD, MUTTI A, FRANCHINI I, LAUWERYS R, ROELS H, BERNARD A, GELPI E, ROSELLO J, HOTTER G AND STOLTE H. (1994). Early urinary markers of target nephron segments as studied in cadmium toxicity. *Kidney Int*., **46**, (suppl. 47), 81–88.

FERENCI P, DRAGOSICS B, DITTRICH H, FRANK H, BENDA L, LOCHS H, MERVIN S, BASE W AND SCHNEIDER B. (1989). Randomized controlled trial of silymarin treatment in patients with cirrhosis of the liver. *J. Hepatol*., **9**, 105–113.

FIELD MJ, BOSTROM TE, SEOW F, GYORY AZ, DYNE M AND COCKAYNE DJ. (1989). Acute cisplatin nephrotoxicity in the rat. Evidence for impaired entry of sodium into proximal tubule cells. *Pflügers Arch*., **414**, 647–650.

HACKE M, SCHMOLL H-J, ALT JM, BAUMANN K AND STOLTE H. (1983). Nephrotoxicity of *cis*-diamminedichloroplatinum with or without ifosfamide in cancer treatment. *Clin. Physiol. Biochem*., **1**, 17–26.

HAHN G AND MAYER A. (1981). Die Mariendistel. *Österr. Apotheker-Zeitung*, **44**, 849–853.

HANNEMANN J AND BAUMANN K. (1988). Cisplatin-induced lipid peroxidation and decrease of gluconeogenesis in rat kidney cortex: different effects of antioxidants and radical scavengers. *Toxicology*, **51**, 119–132.

HAYES DM, CVITKOVIC E, GOLBEY RB, SCHEINER E, HELSON L AND KRAKOFF IH. (1977). High-dose *cis*-platinum diammine dichloride-amelioration or renal toxicity by mannitol diuresis. *Cancer*, **39**, 1372–1381.

HIGBY DJ, WALLACE HJ, ALBERT D AND HOLLAND AF. (1974). Diamminodichloroplatinum II in the chemotherapy of testicular tumours. *J. Urol*., **112**, 100–101.

ISHIKAWA M, TAKAYANAGI Y, SASAKI KI. (1990). Enhancement of cisplatin toxicity by buthionine sulfoximine, a glutathion-depleting agent, in mice. *Res. Comm. Chem. Pathol. Pharmacol.*, **67,** 131–141.

JONES TW, CHOPRA S, KAUFMANN JS, FLAMENBAUM W, TRUMP BF. (1985). *Cis*-diamminedichloroplatinum (II)-induced acute renal failure in the rat. *Lab. Invest.*, **5,** 363–374.

LORENZ D, LÜCKER PW, MENNICKE WH AND WETZELSBERGER N. (1984). Pharmacokinetic studies with silymarin in human serum and bile. *Meth. Find. Exp. Clin. Pharmacol.*, **6,** 655–661.

MATTENHEIMER H, JUNG K, GRÖTSCH H. (1992). Membrane-bound enzymes; Alanine Aminopeptidase. In *Urinary Enzymes in Clinical and Experimental Medicine*. Jung K, Mattenheimer H, Buchardt U, (eds.) Chap. 8.1. Springer Verlag: Berlin.

MAVICHAK V, WONG NLM, QUAMME GA, DIRKS JH AND SUTTON RA. (1985). Studies on the pathogenesis of cisplatin-induced hypomagnesaemia in rats. *Kidney Int.*, **28,** 914–921.

MIDDLETON E AND KANDASWAMI C. (1992). Effects of flavonoids on immune and inflammatory cell function. *Biochem. Pharmacol.*, **43,** 1167–1179.

MIRA L, SILVA M AND MANSO CF. (1994). Scavenging of reactive oxygen species by silibinidihemisuccinate. *Biochem. Pharmacol.* **48,** 753–759.

MUNSHI NC, LOEHRER PJ, WILLIAMS SD, LANGEFELD C, SLEDGE G, NICHOLS VR, ROTH BJ, NEUMAN A AND WALSH WB. (1992). Comparison of N-acetylcysteine and mesna as uroprotectors with ifosfamide combination chemotherapy in refractory germ cell tumours. *Invest. New Drugs*, **10,** 159–163.

OSANTO S, BIKMAN A, VAN HOEK F, STERPH PJ, DELAAT JA AND HERMANS J. (1992). Long term effect of chemotherapy in patients with testicular cancer. *J. Clin. Oncol.*, **10,** 574–579.

PFLEIDERER G, BAIER M, MONDORF AW, STEFANEXCU T, SCHERBERICH JE AND MÜLLER H. (1980). Change in alkaline phosphatase isoenzyme pattern in urine as possible marker of renal disease. *Kidney Int.*, **17,** 242–249.

REECE PA, STAFFORD I, RUSSELL K, KHAN M AND GILL PG. (1987). Creatinine clearance as a predictor of ultrafilterable platinum disposition in cancer patients treated with cisplatin: relationship between peak ultrafilterable platinum plasma levels and nephrotoxicity. *J. Clin. Oncol.*, **5,** 304–309.

SADZUKA Y, SHOIJ T, TAKINO A. (1992). Effects of cisplatin on the activity of enzymes which protect against lipid peroxidation. *Biochem. Pharmacol.*, **43,** 1872–1875.

SAFIRSTEIN R, MILLER P, DIKMAN S, LYMAN N AND SHAPIRO C. (1981). Cisplatin nephrotoxicity in rats: defects in papillary hypertonocity. *Am. J. Physiol.*, **241,** F175–F185.

SAMSON MK, RIVKIN SE, JONES SE, COSTANZI JJ, LOBUGLIO AF, STEPHENS RC, GEHAN EA AND CUMMING GD. (1984). Dose response and dose survival advantage for high versus low-dose cisplatin combined with vinblastine and bleomycin in disseminated testicular cancer. A Southwest Oncology Group Study. *Cancer*, **53,** 1029–1035.

SCHILSKY RL, BARLOCK A AND OZOLS RF. (1982). Persistent hypomagnesaemia following cisplatin chemotherapy for testicular cancer. *Cancer Treat. Rep.*, **66,** 1767–1769.

SKEEHAN P, STORENG R, SCUDIERO D, MONKS A, MCMAHON J, VISTICA D, WARREN JT, BOKESCH H, KENNEY S AND BOYD MR. (1990). New colorimetric cytotoxicity assay for anticancer drug screening. *J. Natl Cancer Inst.*, **82,** 1107–1112.

SONNENBICHLER J AND ZETL I. (1986). Biochemical effects of the flavolignan silibinin on RNA, protein and DNA synthesis in rat livers. In *Plant Flavonoids in Biology and Medicine: Biochemical, Pharmacological and Structure Activity Relationships.* Cody V, Middleton E, Harborn JB (eds.). pp. 319–331. Alan R. Liss Inc.: New York.

SONNENBICHLER J AND ZETL I. (1987). Stimulating influence of a flavolignane derivative on proliferation, RNA synthesis and protein synthesis in liver cells. In *Assessment and Management of Hepatobiliary Disease*. Okolicsanyi I, Cosmos G, Crepadi G (eds.) pp. 265–272. Springer Verlag: Berlin.

SOOSE M. (1994). Properties of silibinin and of antioxidants against adriamycin cytotoxicity in an unicellular eukaryote, *Tetrahymena thermophila. Eur. J. Protistol*, **30,** 394–403.

TAY LK, BREGMAN CL, MASTERS BA AND WILLIAMS PD. (1988). Effect of *cis*-diaminedichloro-platinum (II) on rabbit kidney *in vivo* and on rabbit renal proximal tubule cells in culture. *Cancer Res.*, **48,** 2538–2543.

VALENZUELA A AND GUERRA R. (1985). Protective effects of the flavonoid silyban dehemisuccinate on the toxicity of phenylhdrazine on rat liver. *FEBS Lett*, **81,** 291–294.

VALENZUELA A, LAGOS C, SCHMIDT K AND VIDELA L. (1985). Sylimarin protection against hepatic lipid peroxidation induced by acute ethanol intoxication in rats. *Biochem. Pharmacol.*, **34,** 2209–2212.

WAGNER J, DIESEL P AND SEITZ M. (1974). Zur Chemie und Analytik von Silymarin aus Silybum marianum Gaertn. *Arzneim. Forsch./Drug Res.*, **24,** 466–471.

WERNER-HANSEN S, GROTH S, DAUGAARD G, ROSSING N AND RORTH M. (1988). Long-term effects on renal function and blood pressure of treatment with cisplatin, vinblastine and bleomycin in patients with germ cell cancer. *J. Clin. Oncol.*, **6,** 1728–1731.

WILLIAMS SD, BIRCH R, EINHORN LH, IRWIN L, GRECO FA AND LOEHRER PJ. (1987). Treatment of disseminated germ cell tumors with cisplatin, bleomycin, and either vinblastine or etoposide. *N. Engl. J. Med.*, **316,** 1435–1440.